# Disentangling Safety Culture’s Role in Reducing Cesarean Overuse: Creating a Revised Labor Culture Survey

**DOI:** 10.1089/whr.2024.0023

**Published:** 2024-09-04

**Authors:** Emily White Vangompel, Lavisha Singh, Jin-Shei Lai, Francesca Carlock, Jill Brown, Lisa Kane Low

**Affiliations:** ^1^ Department of Family and Community Medicine, University of Illinois Chicago College of Medicine, Chicago, Illinois, USA.; ^2^ NorthShore University HealthSystem, Evanston, Illinois, USA.; ^3^ Northwestern University, Chicago, Illinois, USA.; ^4^ University of Michigan School of Nursing, Ann Arbor, Michigan, USA.

**Keywords:** cesarean delivery, overuse, labor and delivery, safety culture

## Abstract

**Objective:**

To measure and assess the relationship of patient safety culture to reducing cesarean overuse.

**Study Setting:**

Maternity care hospitals in Michigan.

**Study Design:**

Cross-sectional observational design, combining individual survey data with hospital characteristics using existing databases. Multivariate Poisson regression assessed the associations between survey scores and hospital nulliparous term singleton vertex cesarean rates. Factor analysis determined the scalability of survey items.

**Data Collection Methods:**

Electronic survey distributed at the hospital site level.

**Principal Findings:**

A total of 3091 clinicians from 54 out of 57 eligible hospitals completed the survey. Confirmatory factor analysis demonstrated best fit with a univariate model with two local factors. The new scale encompassing both local factors, including vaginal birth microculture and safety culture, is entitled “Unit Norms.” The safety culture subdomain demonstrated an association with a reduction in hospital cesarean rate [−0.15; 95% CI: −0.27 to −0.04; incident rate ratio (IRR) 0.86], parallel to but lower in magnitude to vaginal birth microculture (−0.18; 95% CI: −0.35 to −0.02; IRR 0.84).

**Conclusions:**

Vaginal birth microculture remains the strongest predictor of cesarean delivery overuse; however, safety culture characteristics, including teamwork, psychological safety, and communication, correlate with lower cesarean delivery rates. Measuring these aspects of hospitals’ culture may be important for other areas of quality improvement initiatives focused on quality and safety.

## Introduction

Efforts to reduce cesarean delivery overuse have met with mixed success.^1–3^ Large statewide perinatal quality initiatives have demonstrated that significant hospital-level reduction is achievable. Yet, many hospitals remain unchanged despite significant external pressure and the use of existing quality improvement tools.^4,5^ Investigation into differences in these “responder” hospitals versus “nonresponders” has found significant differences in attitudes, beliefs, and unit norms surrounding key concepts of supporting vaginal birth, namely low-intervention evidence-based practices such as encouraging early labor at home, use of doulas, and increased nursing in-room time.^6^ Measuring these cultural factors as part of the California Maternal Quality Care Collaborative’s initiative focused on supporting vaginal birth and reducing cesareans has shown that nurses and physicians practicing at the same hospital can have very different attitudes, beliefs, and perceptions of unit norms around vaginal versus cesarean birth.^7^ The personal attitudes of physicians tend to show the strongest association with hospital-level cesarean delivery rates, while this is not true for nurses. Instead, nursing perceptions of collective cultural norms show a stronger association with hospital-level cesarean rates. In addition, greater discordance between nurse and physician groups in terms of attitudes and perceptions is associated with higher cesarean rates.^7^ Staff interactions—functional or dysfunctional—appear essential to cesarean overuse; however, further characterization of the interplay between staff and how it facilitates or hinders quality outcomes is needed.^6^

## Background

Qualitative work with physician and nurse leaders at hospitals participating in quality improvement efforts to support vaginal birth has found that characteristics of solid teamwork, effective team communication, and team member psychological safety are perceived as promoting a hospital’s ability to achieve quality improvement outcomes around cesarean overuse,^6^ characteristics that have been described as patient safety culture.^8^ Patient safety culture, as a concept, has been more often applied to avoiding safety errors rather than supporting a physiological process or preventing overuse of procedures.^9^ The critical domains included in the Agency for Healthcare Research and Quality (AHRQ) model, for example, centrally locate the acknowledgment that an organization’s activities, such as preventing surgical errors or iatrogenic infections, are high risk, and constant vigilance must be exercised to avoid catastrophic safety errors. However, this approach to care is more challenging to apply to the concept of overuse, where the goal is to support physiological processes and limit counterproductive medical intervention.^10^ Unit culture characterized by support for low-intervention evidence-based practices to support vaginal birth (*i.e.,* “vaginal birth microculture”) is associated with reduced cesarean delivery rates^7,11^; however, it is unclear what relationship patient safety culture is likely to have with cesarean overuse.

The Labor Culture Survey (LCS) was initially developed to measure less tangible but essential aspects of labor and delivery units’ cultures to assist in quality improvement efforts being led by state-based perinatal quality collaboratives.^12^ Its use across 70 hospitals in California demonstrated a significant large association with hospital-level nulliparous term singleton vertex (NTSV) cesarean delivery rates.^7^ Patient safety items were not included in its initial development.

### The study

#### Aim and objective

This study aimed to develop patient safety culture survey items that align with the process of supporting vaginal birth, assess their properties in relation to the existing LCS measure of unit norms supportive of vaginal birth, and evaluate their associations with hospital-level cesarean delivery rates.

## Methods

### Design

This was a cross-sectional observational design, combining individual survey data with birth certificate and hospital characteristics, using existing databases. Survey items were administered as part of the larger LCS administration to hospitals in Michigan participating in a statewide initiative to reduce cesarean overuse. Birthing hospitals participating in the collaborative had the option to select administration of the survey or another similar activity as part of completing a menu of quality improvement activities resulting in a summary score and a financial payment to the hospital for their performance. Nurses and providers were recruited to take the survey by email from their local hospital initiative champions. All clinicians (MD/DO, RN, CNM, PA, *etc.*) providing intrapartum care at the hospital were eligible to take the survey. The survey was administered *via* REDCap electronic survey software hosted by the University of Michigan.^13^ The University of Michigan Institutional Review Board determined the study was exempt as quality improvement.

### Labor Culture Survey

The LCS is a measure developed and validated for use in hospital-based quality improvement initiatives to measure baseline individual attitudes, beliefs, and collective unit norms surrounding vaginal versus cesarean birth.^14^ The LCS has been used in several states and by individual hospitals to identify areas with strong cross-disciplinary support, areas with differing disciplinary support, and areas lacking in any support, providing a means to start group conversations, and understanding, and guide quality improvement efforts. The LCS consists of six scales, five measuring individual attitudes, and one measuring collective unit norms around low-intervention evidence-based care to support vaginal birth. LCS scores at the individual hospital level are reported to enable comparisons across hospitals and with regional benchmarks. The LCS measure is significantly associated with NTSV cesarean delivery rates at the hospital level,^7^ and has demonstrated an ability to discriminate between hospitals that can achieve cesarean delivery reduction versus those that cannot.^6^

### Refinement of the LCS

An explanatory mixed-methods study to understand differences between hospitals that successfully decreased their cesarean birth rate compared with those that did not revealed additional domains of unit culture that were not fully captured by the original LCS.^6^ To fill these gaps, a multidisciplinary group of clinicians and health service researchers completed additional enhancements to the LCS. An additional nine items were developed based on qualitative semistructured interviews and focus groups with labor and delivery leaders and clinicians at hospitals that had previously participated in a quality improvement initiative to reduce cesarean overuse. These qualitative methods and analyses have been previously described.^6^ Newly added items included leadership involvement, communication, psychological safety, personal responsibility/ownership of safety outcomes, and collaborative team structure in relation to supporting vaginal birth. Some items were designed specifically for labor and delivery nurses (nurses), yet some were only for physicians and midwives (providers). Items were created based on qualitative findings, not on an *a priori* definition of safety culture. These new items were added as part of the survey launch in Michigan.

### Covariates

Cesarean births performed in a low-risk population, defined as being nulliparous, term (>37 weeks) with a singleton fetus in the vertex presentation (NTSV), and NTSV total births were obtained from the 2019 birth certificate data from the Michigan Department of Health and Human Services along with other hospital-level covariates, including annual birth volume, percent maternal population with Medicaid (% Medicaid), percent maternal population with a body mass index over 30 (% BMI >30), and percent maternal population older than 35 years (% Age >35). The Michigan Alliance for Innovation in Maternal Health provided hospital geographic location (rural/urban-suburban), and nursery acuity level was provided by the Michigan Department of Health and Human Services.

### Statistical analyses

We compared clinician respondent characteristics and participating hospital characteristics among the top quartile (NTSV cesarean rate <24%) and other hospitals using the chi-squared test for categorical variables and Student’s *t-test* or Wilcoxon rank sum test for continuous variables. We evaluated the scalability of the original LCS items and newly added items by examining essential unidimensionality using bifactor analyses, consisting of one general factor representing *Labor Culture* and two local factors (*i.e.,* subdomains) representing *Vaginal Birth Microculture* and *Safety Culture*, respectively. We started with an exploratory bifactor analysis to review whether items loaded on the designated local factor (loading >0.4). We then applied confirmatory bifactor analysis using the following criteria: comparative fit index (CFI) >0.90, root mean square error of approximation (RMSEA) <0.1, residual correlations between item-pairs <0.15, and item loadings to the general factor >0.3. In addition, we calculated McDonald’s omega_hierarchical_(ω_h; criterion:_ >0.70) to support unidimensionality of the general factor. We evaluated individual items and overall scale association with hospital-level 2019 NTSV cesarean delivery rate using multivariate Poisson regression. We used PROC GENMOD with a log link function and the distribution was specified as Poisson. Hospital 2019 NTSV birth volume was used as the offset term. Scale Poisson models were adjusted for hospital-level demographics, including geographic location, nursery acuity level, and maternal obstetric risk categories (BMI and age). All statistical analyses were performed using SAS version 9.4 (SAS Institute Inc., Cary, NC, USA).

## Findings

### Participants

Of the potential participating hospitals, 57 had selected the LCS option. Of the 57 hospitals, 54 achieved the minimum 30% response rate necessary from their hospital clinicians, with representation from obstetricians and nurses required for analysis. The resulting sample then was 3,091 respondents. Respondents reflected the labor and delivery workforce in Michigan, which is majority female and White, non-Hispanic. Neither individual respondents nor hospital characteristics differed by cesarean delivery performance categories (Table 1).

**Table 1.** Characteristics of Clinician Respondents in Participating Michigan Hospitals by Cesarean Delivery Overuse Performance

	Overall	Top quartile^a^	≥26th percentile^a^	
	n (%)	n (%)	n (%)	
	3091 (100)	616 (19.9)	2475 (80.1)	*p* value
Role				0.09
Labor and delivery nurse	1690 (54.7)	341 (55.4)	1349 (54.5)	
Obstetrician	499 (16.1)	93 (15.1)	406 (16.4)	
Certified nurse midwife	141 (4.6)	16 (2.6)	125 (5.1)	
Family medicine physician	75 (2.4)	13 (2.1)	62 (2.5)	
Anesthesiologist	198 (6.4)	38 (6.2)	160 (6.5)	
Nurse educator	44 (1.4)	14 (2.3)	30 (1.2)	
Nurse manager/director	81 (2.6)	20 (3.2)	61 (2.5)	
Resident	239 (7.7)	53 (8.6)	186 (7.5)	
Other	124 (4)	28 (4.5)	96 (3.9)	
Residency specialty				0.30
OB/GYN	186 (77.8)	44 (83)	142 (76.3)	
Family medicine	53 (22.2)	9 (17)	44 (23.7)	
Race/ethnicity				0.11
White (non-Hispanic)	2657 (86)	533 (86.5)	2124 (85.8)	
Black/African American	117 (3.8)	18 (2.9)	99 (4)	
Hispanic/Latino	48 (1.6)	16 (2.6)	32 (1.3)	
Asian/Pacific Islander	92 (3)	16 (2.6)	76 (3.1)	
American Indian/Alaska Native	15 (0.5)	5 (0.8)	10 (0.4)	
Prefer not to say	116 (3.8)	22 (3.6)	94 (3.8)	
Other	46 (1.5)	6 (1)	40 (1.6)	
Gender				0.82
Male	372 (12)	74 (12)	298 (12)	
Female	2662 (86.1)	528 (85.7)	2134 (86.2)	
Nonbinary/third gender	3 (0.1)	1 (0.2)	2 (0.1)	
Prefer not to say	54 (1.7)	13 (2.1)	41 (1.7)	
Residency year				0.95
PGY-1	68 (28.5)	15 (28.3)	53 (28.5)	
PGY-2	60 (25.1)	12 (22.6)	48 (25.8)	
PGY-3	67 (28)	15 (28.3)	52 (28)	
PGY-4+	44 (18.4)	11 (20.8)	33 (17.7)	
Years of training, median (IQR)	15.0 (6.0–25.0)	14.0 (7.0–24.0)	15.0 (6.0–25.0)	0.70
Years worked, median (IQR)	9.0 (4.0–20.0)	10.0 (4.0–20.0)	9.0 (4.0–20.0)	0.36
% Maternal age >35	5.6 ± 3.1	4.4 ± 2.1	5.9 ± 3.3	0.15
% Maternal BMI >30	27.8 ± 5.0	29.7 ± 5.6	27.2 ± 4.8	0.28
% Publicly insured	40.2 ± 16.7	44.4 ± 13.6	38.9 ± 17.6	0.34
Teaching hospital				0.92
Yes	41 (75.9)	10 (76.9)	31 (75.6)	
No	13 (24.1)	3 (23.1)	10 (24.4)	
NICU				0.53
No	25 (46.3)	7 (53.8)	18 (43.9)	
Yes	29 (53.7)	6 (46.2)	23 (56.1)	
Rural/urban–suburban				0.78
Urban–suburban	39 (72.2)	9 (69.2)	30 (73.2)	
Rural	15 (27.8)	4 (30.8)	11 (26.8)	
Average annual delivery volume				0.32
Less than 1000	5 (9.3)	0 (0)	5 (12.2)	
1000–2499	49 (90.7)	13 (100)	36 (87.8)	
2500 or more	0 (0)	0 (0)	0 (0)	
State of Michigan Prosperity Regions				0.18
1. Upper Peninsula	3 (5.6)	1 (7.7)	2 (4.9)	
2. Northwest	2 (3.7)	0 (0)	2 (4.9)	
3. Northeast	3 (5.6)	1 (7.7)	2 (4.9)	
4. West	7 (13)	3 (23.1)	4 (9.8)	
5. East Central	4 (7.4)	0 (0)	4 (9.8)	
6. East Michigan	6 (11.1)	1 (7.7)	5 (12.2)	
7. South Central	1 (1.9)	1 (7.7)	0 (0)	
8. Southwest	5 (9.3)	3 (23.1)	2 (4.9)	
9. Southeast	5 (9.3)	0 (0)	5 (12.2)	
10. Detroit Metro	18 (33.3)	3 (23.1)	15 (36.6)	

^a^ Top quartile hospitals in this sample had NTSV cesarean rates <24%. “≥26th Percentile” hospitals include all hospitals not included in the top quartile group.

NTSV, nulliparous term singleton vertex; MD, physicians; RN, nurses; PGY, postgraduate year; IQR, interquartile range; BMI, body mass index; NICU, neonatal intensive care unit.

**FIG. 1.**
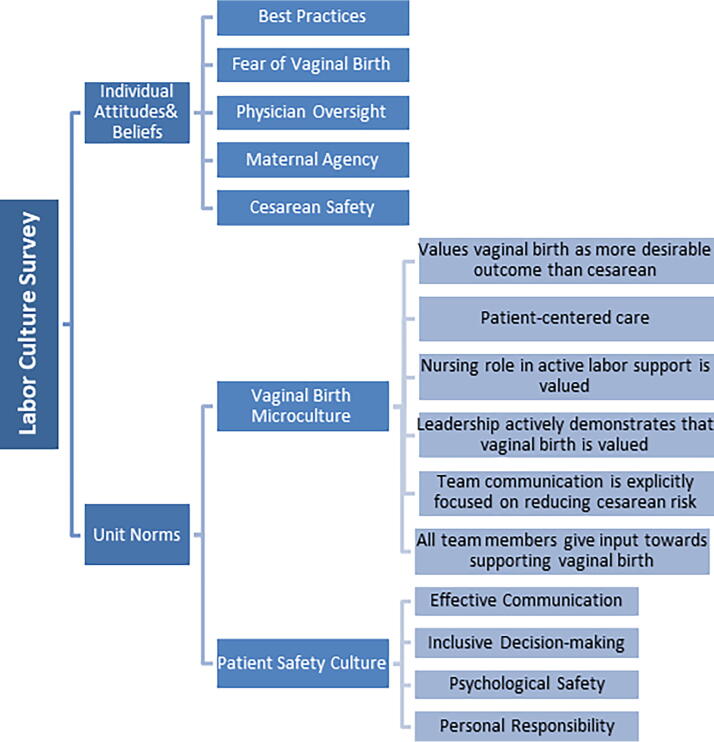
Conceptual model of Labor Culture as measured by the Revised Labor Culture Survey.

Refinement of the newly introduced patient safety subscale focused on the nine new items written to cover critical gaps raised during focus groups and interviews that the existing LCS did not already cover. Two items specific to RNs (“I feel supported to advocate on behalf my patients to maximize their chance of vaginal birth”) and physicians (“It is my responsibility to maximize my patients’ chance of having a vaginal birth”) were combined as both captured the same content but to different clinicians. Eight items were retained after factor analyses (4 unique, 2 provider-specific, and 2 nurse-specific matched items). A single question asked of only nurses was found to have a low correlation coefficient (<0.03) with other items on the scale, and thus, it was dropped. In terms of dimensionality evaluation, three items were found to not load (<0.4) to the general factor and/or were not associated with either local factor. The study team reviewed the item content and decided to remove these three items. Of these three, one referred to patient knowledge level and one referred to interactions with doulas, both important contributors to supporting vaginal birth, but conceptually less related to interprofessional interactions between staff. The third question had strong face validity with the vaginal birth microculture local factor but may have reflected personal attitudes more than perceptions of unit norms, as it asked for degree of agreement with, “There are too many cesarean births performed on my L&D unit.” Sufficient unidimensionality was supported to the remaining 11 items: CFI = 0.981, RMSEA = 0.078, all residual correlations <0.15, loadings to the general factor were >0.3, and ω_h; criterion_ was 0.74. Subsequently, a single summation score representing overall Unit Norms or two subscale scores representing Vaginal Birth Microculture and Safety Culture can be used. The final conceptual model for the revised LCS is seen in FIG. 1.

Individual Safety Culture items’ associations with hospital-level NTSV cesarean delivery rates demonstrated that each item was inversely associated with lower cesarean delivery rates (Table 2). The Safety Culture subdomain showed an adjusted association of −0.15 [95% CI: −0.27 to 0.04; incident rate ratio (IRR) 0.86], indicating that for every 1 point of mean increased agreement that a hospital has a strong patient safety culture, NTSV cesarean rate decreased by a relative 14%. The revised Vaginal Birth Microculture subdomain demonstrated an adjusted association of −0.18 (95% CI: –0.35 to −0.02; IRR 0.84), indicating that for every 1 point of mean increased agreement that a hospital’s unit norms support vaginal birth, NTSV cesarean rate decreased by a relative 16%. The two subscales were correlated at the facility level (correlation coefficient = 0.78), but there was no statistically significant interaction effect between the two subscales in regard to their effects on NTSV cesarean rate (*p* = 0.79). The associations between each subscale and the model covariates are in Supplementary Table S1, and the associations between model covariates and NTSV cesarean rates are in Supplementary Table S2. No covariates demonstrated a significant association with either of the subdomains or with NTSV cesarean delivery rates, consistent with prior studies.^7^ Qualitative data suggest that there may be unmeasured confounding associated with the perceived risk environment of a particular hospital unit and the attitudes, beliefs, and norms expressed by clinicians in that environment^6^; thus, we chose to be conservative and adjust for these covariates in our multivariate modeling.

**Table 2.** Adjusted^a^ Associations Between Hospitals’ Mean Score on the Safety Culture Scale, Vaginal Birth Microculture Scale, and Individual Safety Culture Scale Items and 2019 NTSV Cesarean Delivery Rate

	Unadjusted		Adjusted	IRRs	
**Subscale/Items**	**Estimate (95% CI)**	*p* Value	**Estimate (95% CI)**	**e^β^**	*p* Value
Local factor 1 (Vaginal Birth Microculture) (q13a^b^ q13d^b^ q13g^b^ q13h^b^ q13i^b^ q13j q13k)	−0.15 (−0.30, −0.003)	**0.045**	−0.18 (−0.35, −0.02)	0.84	**0.028**
13j: In my unit, nurses, physicians, and midwives communicate frequently during a patient’s labor to discuss supporting vaginal birth and reducing cesarean risk	−0.11 (−0.21, −0.01)	**0.048**	−0.12 (−0.23, −0.01)	0.89	**0.044**
13k: In my unit, nurses, physicians, and midwives have equal input in management decisions that may affect a patient’s chance of having a vaginal versus cesarean birth	−0.12 (−0.23, −0.02)	**0.021**	−0.15 (−0.26, −0.03)	0.86	**0.011**
Local factor 2 (Safety Culture) (q13l rq13e^b^ q9bd q9ac)	−0.13 (−0.23, −0.02)	**0.020**	−0.15 (−0.27, −0.04)	0.86	**0.010**
13l: In my unit, all clinicians-nurses, physicians, and midwives feel safe and encouraged to speak up if a patient’s chance of having a vaginal birth may be negatively affected by my management decisions	−0.11 (−0.22, −0.00)	**0.042**	−0.14 (−0.26, −0.02)	0.87	**0.022**
13i: My hospital leadership is actively engaged in making change in our unit to support vaginal birth and reduce primary cesarean sections (*i.e.,* adequate resources, supplies, staffing, education)	−0.08 (−0.19, 0.03)	0.178	−0.13 (−0.26, −0.01)	0.88	**0.045**
9ac: MD-It is my responsibility to maximize my patients’ chance of having a vaginal birth; RN-I feel supported to advocate on behalf of my patients to optimize their chance of vaginal birth	−0.12 (−0.23, 0.00)	0.056	−0.14 (−0.26, −0.01)	0.87	**0.034**
9bd: MD-I encourage my hospital’s nurses to play an active role in helping me maximize my patients’ chance of having a vaginal birth; RN-The physicians I work with encourage my input when making patient management decisions	−0.10 (−0.20, −0.01)	**0.031**	−0.13 (−0.24, −0.03)	0.88	**0.016**

Bolded text indicates statistical significance, with p<0.05.

^a^ Adjusted for hospital annual birth volume, geographic location, nursery acuity level, maternal % BMI >30, maternal % age >35 y, and maternal % Medicaid as primary insurance.

^b^ These items were previously published in the original validation article^12^ as supporting information and can be accessed here: https://onlinelibrary.wiley.com/doi/10.1111/birt.12406

NTSV, nulliparous term singleton vertex; MD, physicians; RN, nurses; IRR, incident rate ratio.

## Discussion

In this large sample, representing the majority of birthing hospitals across the state of Michigan, there was a univariate measure of unit norms, encompassing one unidimensional domain, aka Unit Norms, with two subdomains, Safety Culture and Vaginal Birth Microculture. Although one single Unit Norms score can be produced, we took a further step to evaluate the relationship at the subdomain level and found that Safety Culture and Vaginal Birth Microculture were significantly associated with hospitals’ NTSV cesarean rates. Vaginal Birth Microculture demonstrated a slightly larger magnitude of association compared with Safety Culture. The Vaginal Birth Microculture subdomain measures collective norms around addressing and preventing the cascade of intervention that leads to overuse of cesarean birth, which, although related, is not the same as creating a safety culture to prevent safety errors. Content captured by these two subdomains appears to be related and complementary when it comes to cesarean delivery overuse, which was also supported by our unidimensionality finding. This work supports the importance of patient safety culture in quality improvement initiatives, where it has been found that hospitals that more easily implement changes have a stronger patient safety culture.^15^ Likely this also signifies a positive level of provider engagement and positive team-based communications. However, it also underscores the importance of unit norms that are unique to reducing overuse of cesareans, namely, supporting and valuing physiological birth through low-intervention evidence-based norms.

This distinction between what is considered a good “Culture of Safety” in the majority of inpatient settings versus a “Culture Supportive of Vaginal Birth” may be partially due to the difficulty in defining overuse of a medical procedure such as cesarean as a patient safety issue.^16^ It has been argued that overuse should be viewed as equally harmful to patients as adverse events, and should have equal representation at the quality review committee table to create impetus for change.^17^ Underuse or misuse (*e.g.,* postpartum hemorrhage mismanagement) as opposed to overuse (*e.g.,* cesarean delivery overuse) is more often the focus of hospital perinatal quality review committees. Few hospitals expend the same amount of effort in reviewing cases of potential cesarean overuse, which have immediate applications for practice improvement, compared with serious adverse events such as stillbirth, which may have few actionable hospital-based improvements. This by no means equates the level of harms between cesarean overuse and catastrophic maternal or fetal outcomes; however, a system that looks only at the most recent pregnancy to determine how a death from severe maternal hemorrhage due to placenta accreta could have been avoided fails to address the likely root cause of such a catastrophic outcome—the first cesarean delivery.^18^ This example demonstrates the challenge of only looking downstream at the outcomes instead of focusing upstream where prevention may be more possible.

This study had limitations, including its use of a convenience sample; however, given the extremely high participation rate from hospitals across the state of Michigan, including rural, community, teaching, and urban hospitals, it is reasonable to consider this sample representative of the state. The range of providers represented in the survey also mirrors those across the state with all but 2 included hospitals achieving a 50% response rate or greater and having at least 30% of the type of providers (*e.g.,* physicians, nurses, midwives, residents) represented for each hospital. In addition, the Safety Culture scale items may not encompass all important features of Patient Safety Culture as defined by the AHRQ and others, and future work should consider comparing the longer AHRQ Culture of Safety measure to the LCS and Safety Culture scale within the same setting.

## Conclusion

Hospitals expend significant time and resources investing in the attainment of markers of safety and quality, such as Magnet Hospital status, LeapFrog Hospital Safety Grade, and Joint Commission-accreditation; however, it is possible that these goals leave out important components of quality that are unique to preventing and addressing overuse of a practice or intervention. Future work should examine the relationship between patient safety culture, overuse, and adverse events at hospitals to better define needs and develop tools for quality improvement.

## Supplementary Material

Supplementary Table S1

Supplementary Table S2

## Abbreviations Used

AHRQ: Agency for Healthcare Research and Quality
BMI: body mass index
CFI: comparative fit index
CNM: certified nurse midwife
LCS: Labor Culture Survey
MD/DO: physician
NTSV: nulliparous, term, singleton, vertex
PA: physician assistant
RMSEA: root mean square error of approximation
RN: nurse
