# Supplementary material for: Disentangling Safety Culture’s Role in Reducing Cesarean Overuse: Creating a Revised Labor Culture Survey
Source: Womens Health Rep (New Rochelle). 2024 Sep 4;5(1):594–601. doi: 10.1089/whr.2024.0023 (PMC11462421; doi:10.1089/whr.2024.0023)
Supplement: Supplementary Table S1 [file whr.2024.0023_SupplementaryTable1.pdf]

Supplementary Table 1. Associations between model covariates and NTSV cesarean rates for each Unit Norms sub-scale

Parameters	Estimate (95% CI)	p-value
**Local factor 1 (Vaginal Birth Microculture)**	-0.18 (-0.35 , -0.02)	0.028
**% Maternal BMI >30**	-0.003 (-0.010 , 0.005)	0.473
**% Maternal Age >35**	-0.004 (-0.018 , 0.011)	0.631
**% Publicly Insured**	-0.001 (-0.004 , 0.001)	0.272
**NICU (yes vs no)**	0.001 (-0.077 , 0.079)	0.988
**Average Annual delivery volume**	-0.000 (-0.0001 , 0.000)	0.655
**Rural vs urban-suburban**	0.004 (-0.100 , 0.108)	0.938
**Local factor 2 (Safety Culture)**	-0.15 (-0.27 , -0.04)	0.010
**% Maternal BMI >30**	-0.003 (-0.010 , 0.004)	0.433
**% Maternal Age >35**	0.001 (-0.014 , 0.016)	0.928
**% Publicly Insured**	-0.001 (-0.003 , 0.001)	0.396
**NICU**	0.002 (-0.076 , 0.080)	0.961
**Average Annual delivery volume**	0.000 (-0.0001 , 0.000)	0.434
**Rural vs urban-suburban**	0.011 (-0.091 , 0.114)	0.828

Supplementary Table 2. New items and model covariates

	Local factor 1 Spearman corr coefficients	p-value	Local factor 2 Spearman corr coefficients	p-value
**% Maternal BMI >30**	0.03	0.83	-0.03	0.81
**% Maternal Age >35**	-0.12	0.39	0.03	0.85
**% Publicly Insured**	-0.25	0.07	-0.09	0.51
**Average Annual delivery volume**	-0.09	0.51	-0.09	0.52
**NICU**	mean ± std dev	0.43	mean ± std dev	0.57
No	2.96 ± 0.27		2.84 ± 0.28	
Yes	2.92 ± 0.17		2.82 ± 0.26	
**Rural/urban-suburban**		0.18		0.26
Urban-suburban	2.91 ± 0.17		2.81 ± 0.27	
Rural	3.00 ± 0.31		2.88 ± 0.27	
